# Extraction of rapeseed cake oil using subcritical R134a/butane: Process optimization and quality evaluation

**DOI:** 10.1002/fsn3.1209

**Published:** 2019-09-26

**Authors:** Tingting Guo, Chuyun Wan, Fenghong Huang

**Affiliations:** ^1^ Oil Crops Research Institute Chinese Academy of Agricultural Sciences Wuhan China; ^2^ Hubei Key Laboratory of Lipid Chemistry and Nutrition Wuhan China; ^3^ Oil Crops and Lipids Process Technology National & Local Joint Engineering Laboratory Wuhan China

**Keywords:** butane extraction, extraction oil quality, rapeseed cake, response surface methodology, subcritical R134a

## Abstract

The optimal extraction conditions of rapeseed cake oil using subcritical R134a/butane were established by response surface methodology. The quality of subcritical R134a/butane extraction oil (SRBEO) was compared with supercritical CO_2_ extraction oil (SCO_2_EO) and hexane extraction oil (HXEO). The results showed the highest extraction yield obtained by subcritical R134a/butane in the condition of R134a‐butane ratio of 1.5 kg/kg, at 45°C for 50 min. Compared with SCO_2_EO and HXEO, the extraction yield and β‐carotene content of SRBEO (87.76%, 357.21 μg/100g) were the highest. The content of phospholipids and canolol in SRBEO (3.01 mg/g, 118.51 mg/100 g) was higher than SCO_2_EO (not detected, 95.82 mg/100 g) and less than HXEO (25.78 mg/g, 131.85 mg/100 g). The tocopherols in SRBEO were equivalent to SCO_2_EO but phytosterol content of SRBEO (560.19 mg/100 g) was less than SCO_2_EO (591.40 mg/100 g). For fatty acids, the three extraction oils had slight difference. Thus, subcritical R134a/butane extraction appeared to be feasible for rapeseed cake oil extraction.

## 1 INTRODUCTION

Rapeseed is a cruciferous seed, appearing round or oval. Rapeseed is planted wildly in China, and the planting area accounts for more than 40% in all oil crops and the oil yield about 30% in total oil yield. At present, approximately 90% of rapeseed varieties are double‐low (low erucic acid and low glucosinolates) oilseed rape (Yang et al., 2014). Double‐low rapeseed oil is high‐quality edible oil with low erucic acid content and reasonable fatty acid composition (Li et al., 2016). Rapeseed meal as by‐product after oil extraction remains much oil, with approximately 12%–15%, which is unbenefited for manufacturers. Moreover, rapeseed cake is rich in tocopherols, polyphenols, phytosterols, and phospholipids (SzydlOwska‐Czerniak, Amarowicz, & Szlyk, 2^2010^), which possess important biological and chemical properties. Therefore, it is of great significance to the further development and utilization of oil and minor components in rapeseed cake.

Generally, residue oil in cake is extracted by hexane (Xu, Han, Zhou, Wu, & Ding, 2016). Hexane extraction has the advantages of large processing capacity and low production cost. However, the oil extraction contains much residual solvent. In addition, the high‐temperature extraction and desolvation process will destroy the thermal sensitive substances in the oil. At the same time, it also brings environmental problems such as waste heat dissipation and emission (Campbell et al., 2011). Supercritical CO_2_ extraction is also commonly used in the extraction of oils and fats, which has the advantages of low cost, nontoxicity, and environmental friendliness. The density, viscosity, and diffusion coefficient of CO_2_ can be controlled by adjusting the temperature and pressure of SCO_2_, achieving the aim of improving the extraction rate or selectivity of oil and fat (Cvjetko et al., 2012; Jokić, Bijuk, Aladić, Bilić, & Maja, 2016; Li, Xia, Vazquez, & Song, 2017). However, the pressure of supercritical extraction is high and the equipment is expensive (Sahena et al., 2009), which have kept the technology from being widely used in industries. Subcritical fluid extraction (SFE) is a new separation technology developed after supercritical fluid extraction. It guarantees quality and productivity, and enables industrialization. Thus, for the rapeseed meal, SFE is a better choice to maintain oil quality (Russin, Boye, Arcand, & Rajumohamed, 2011). Several solvents can be used as subcritical fluids to extract plant oil or animal lipid, such as propane, butane, dimethyl ether (DEM), and 1,1,1,2‐tetrafluoroethane (R134a). Teixeira, Ghazani, Corazza, Marangoni, and Ribani (2018) investigated the extraction of sapucaia oil with subcritical propane and obtained 93.38% oil with good oxidative stability. Sun et al. (2018) studied antarctic krill lipid using subcritical butane extraction. Results indicated that the lipid recovery rate was 81.2%, and most astaxanthin (248.4 mg/kg) remained in product. Noteworthily, R134a presents low toxicity, inertia, nonflammability, and mild critical conditions. R134a is suitable and effective to extracting components such as procyanidins (Tan, Zhu, & Feng, 2018), polyphenols, β‐carotene (Mohd Setapar, Khatoon, Ahmad, Yunus, & Zaini, 2014; Mustapa, Manan, Azizi, Norulaini, & Omar, 2009), and polycyclic aromatic hydrocarbons (Shi, Qiao, & Feng, 2016). Nevertheless, some solutes (such as fatty acids and triglycerides) habited a slight solubility even in hot R134a. Modification by adding a small amount of n‐butane or dimethyl ether as gaseous cosolvents with boiling points similar to that of R134a could improve the ability for nonpolar solutes. At present, there are few reports about the binary mixtures of R134a and butane as extraction solvents.

Compared with rapeseed cake, large quantities of solvents are required during the subcritical extraction of rapeseed, which increases the cost. Therefore, most of the oil can be extracted firstly by low‐temperature pressing, and the residual oil in rapeseed cake can be obtained by subcritical extraction. In this experiment, we adopted the binary mixture of R134a and butane as an extractant, and Box–Behnken response surface method was used to design and optimize the subcritical extraction process parameters. Furthermore, the quality characteristics of the extraction oil were compared with supercritical CO_2_ extraction oil and hexane extraction oil.

## 2 MATERIALS AND METHODS

### 2.1 Materials

Rapeseed cake (moisture content, 4.80% and residual oil content, 12.79%) was obtained by low‐temperature pressing of Zhongshuang 11 double‐low rapeseed in pilot plant of Oil Crop Research, Chinese Academy of Agricultural Sciences.

### 2.2 Subcritical R134a/butane extraction

Supercritical fluid extraction was executed using a PLE‐5L subcritical fluid extraction system (Henan Subcritical Extraction Biological Technology Co., Ltd). Rapeseed cake was placed in the extraction vessel and sealed with cover. At first, the vacuum pump was turned on to reduce the pressure in extraction vessel, separation vessel, and measuring vessel to −0.01 MPa. Subsequently, the static extraction ran. The ratio of material to solvent (the mixture of R134a and butane, and the mass ratio was 6:1, 6:2, 6:3, 6:4, 6:6, kg: kg) was 1:8 (w/v). Following that, the oily solution was introduced into the separation vessel. The R134a/butane was compressed by compressor, and the vacuum pump was opened for further recovery until the pressure of the three vessels to −0.01 MPa. Extract oil was collected from the separator vessel and centrifuged at 4,863 *g* for 10 min, and the upper oil was stored at 4°C for analysis.

Response surface methodology with Box–Behnken design (BBD) was used to optimize the process parameters of subcritical fluid extraction. The mass ratio of R134a and butane (A), extraction temperature (B), and extraction time (C) were independent variables, and extraction yield was dependent variable. According to single‐factor experiment results, the extraction temperature is set at 25–45°C and the extraction time is 10–50 min. The codes and level values of independent variable are shown in Table 1. The experiment was carried out in a random order. Response surface plots were made by Design Expert (DE), and the relationship between the factors was analyzed.

**Table 1:** Codes and level values for independent variable

Factor	Code	−1	0	1
R134a‐butane ratio (kg/kg)	*A*	1 (6:6)	2 (6:3)	3 (6:2)
Extraction time (min)	*B*	10	30	50
Extraction temperature (°C)	*C*	25	35	45

The extraction yield was calculated as:

$Y=\frac{m_{0}-m_{1}}{m_{0}}\times 100$.

where *Y* was the extraction yield (%), and *m*_0_ and *m*_1_ were masses of oil in raw materials and extracted cake (g), respectively.

### 2.3 Supercritical CO_2_ extraction

Supercritical CO_2_ extraction was performed using a HA221‐50–06 supercritical fluid extraction system (Hua'an Supercritical Fluid Extraction Corp.) equipped with high purity CO_2_ (99.99%). 150 g cake was weighted in sample vessel and extracted. The extraction temperature, pressure, time, and the flow rate of CO_2_ were set at 40°C, 28 MPa, 50 min, and 18 L/hr. The extraction oil was collected from separation I and II and centrifuged at 4,863 *g* for 10 min; the upper oil was stored at 4°C for analysis.

### 2.4 Hexane extraction

Hexane extraction of rapeseed cake was carried out in two steps. 150 g of cake and 1,200 ml of hexane solvent were homogenized and held for 50 min in water bath at 45°C. Then, the extracted oil was collected by solvent recover by vacuum evaporation. The extracted oil was collected and stored at 4°C for analysis.

### 2.5 Determinations

#### 2.5.1 Residual oil content

The residual oil content in rapeseed cake was determined according to the ISO659.2009, which involves the gravimetric analysis with analytical grade petroleum ether in a Soxhlet apparatus (B‐811; Buchi Labortechnik AG) for 8 hr.

#### 2.5.2 Physicochemical characteristics

Viscosity measurements were determined by the method of Chouaibi et al. (2012) with a few modifications, and oil viscosity was run using a strain‐controlled rheometer (AR 2000, TA Instruments, Ltd.). The temperature was maintained at 25°C with a Peltier plate (±0.1°C). And shear rate was in the range of 0.1–100/s with a cone geometry, whose diameter, cone angle, and gap between cone and plate were 40 mm, 2°, and 57 μm, respectively.

A Lovibond tintometer (WSL‐2A, Suoguang Electric Technology Co., Ltd.) was used to determine the color of the rapeseed cake oil. Acid value, peroxide value, refractive index, saponification value, and iodine value were determined by ISO 660.2009, ISO 6320.2000, ISO 3960.2007, ISO 3657.2013, and ISO 3961.2013, respectively.

#### 2.5.3 Fatty acid

Oil samples were methylated using sodium methoxide as reported by Teixeira et al. (2018) and determined by Agilent 7890A gas chromatography system (Agilent) equipped with HP‐INNOWAX capillary column (30 m × 0.32 mm, 0.25 μm, Agilent Corp). The injector and detector temperatures were both 260°C. The flow rate of the carrier gas N_2_ with a split ratio of 80:1 was set at 1.5 ml/min. The oven temperature was programmed as follows: helding the initial temperature of 210°C for 9 min, followed by an increase to 350°C at the rate of 20°C/min for 10 min. The injection volume was 1 *μ*L. The fatty acid composition of samples was analyzed by comparing the retention time of standard fatty acids. The relative ratio of fatty acids was calculated by normalization method.

#### 2.5.4 Phospholipids

Phospholipid content of oil samples was analyzed according to the method of Xu et al. (2016). 3 g of oil was accurately weighed and 0.1 g of zinc hydroxide was added to a crucible. The sample was carbonized before being completely ashed in a muffle furnace at 575°C for about 2 hr. The ash was dissolved in 10 ml of 50% HCl solution and heated to a slight boiling point and held for 5 min. The solution was cooled and filtered into a 100‐mL volumetric flask. 50% (w/v) KOH solution was used to neutralize to turbidity. 50% (v/v) HCl solution was slowly added to completely dissolve the precipitation. The solution was diluted with distilled water to the scale. 10 ml of test solution was pipetted into a 50‐mL colorimetric tube. 8 ml of hydrazine sulfate solution (0.0015%, w/v) and 2 ml of sodium molybdate solution (2.5%, w/v) were added. The tube was heated in a boiling water bath for 10 min and cooled to room temperature. The solution was diluted with distilled water to the scale and determined against a blank using a DU 800 UV/Visible Spectrophotometer (Beckman Coulter Inc., USA) at 650 nm.

#### 2.5.5 Phospholipid compositions

A 4 g (exact to 0.0001 g) of oil was weighed into a 100‐mL test tube, and 50 ml of trichloromethane was added. 10 ml solution was transferred into an activated amino silica gel solid‐phase extraction column. The column was then eluted with 2.0 ml of trichloromethane–isopropanol mixed solution and 3 ml of acetic acid–ether mixed solution, respectively. Phospholipids were eluted with 3 ml methanol. The eluent was evaporated by rotary evaporator at 45°C. The residue was dissolved in 10 ml hexane–isopropanol–acetic acid (8:8:1, v/v/v) and centrifuged for 5 min at 4,863 × g. The supernatant was separated for further analysis. High‐performance liquid chromatography (HPLC) (LC‐20A; Shimadzu Corp) on a SIL60 column (250 × 4.6 mm, 5 μm; GL Sciences Inc.) was performed at a flow rate of 1 ml/min with hexane–isopropanol–acetic acid (8:8:1, v/v/v) as mobile phase. The injection volume was 1 *μ*l, and the detection wavelength was 205 nm.

#### 2.5.6 Canolol

Canolol content in oil was determined by the method of Yang et al. (2014) with a few modifications. Oil sample of 1.25 g was mixed with 1.5 ml of 80% methanol solution and 1.5 ml of hexane for three times. The extracts were combined and through a 0.22‐*μ*m nylon filter. The analysis was carried out with ACQUITY ultra‐performance liquid chromatography (UPLC) (Waters) equipped with photodiode array (PDA) detector and Waters ACQUITY BEH Shield RP18 (100 × 2.1 mm, 1.7 μm, Waters). The column temperature was 30°C, and injection volume was 3 *μ*l. Extracts were eluted with 2% acetic acid (w/w) (mobile phase A) and 100% methanol (mobile phase B) at the flow of 0.21 ml/min. The gradient elution was as follows: 5%–25% B (7.40 min), 25%–29% B (2.67 min), 29%–36% B (6.66 min), 36%–45% B (6.67 min), 45%–65% B (2 min), 45%–65% B (2 min), 65%−5% B (2 min), and 5% B (2.67 min). The detection wavelength was 270 nm.

#### 2.5.7 *β*‐Carotene

*β*‐Carotene content in oil was measured according to the method of Gimeno et al. (2000) with a few modifications. A 2 g of oil was saponified thoroughly with 1 g of ascorbic acid, 75 ml of absolute ethanol, and 25 ml of potassium hydroxide solution (1 g/ml) and incubated at 60°C for 30 min. The target ingredient was extracted twice with 100 ml of petroleum ether. The organic layer was recovered and dissolved with 5 ml of dichloromethane. The chromatographic analysis was carried out using high‐performance liquid chromatography (HPLC) (LC‐20A, Shimadzu Corp) on a C30 column (150 × 4.6 mm, 5 μm; GL Sciences Inc.) with a gradient elution mode: 0%– 40% B (methyl tertiary butyl ether) (15 min), 41%–80% B (3 min), 80% B (3 min), 80%–100% B (1 min), and 0%–100% A (methyl–acetonitrile–ultrapure water, 73.5:24.5:2, v/v/v) (2 min). The elution was implanted at a flow rate of 1.0 ml/min, the injection volume was 1 *μ*L, and the detection wavelength was 450 nm.

#### 2.5.8 Tocopherols

The content of tocopherols in oil was determined according to AOCS Official Method Ce 8–89 with slight modifications. A 2 g (accuracy 0.0001g) of samples was dissolved in hexane and through a 0.22‐*μ*m polytetrafluoroethylene filter. The treated samples (20 *μ*l) were analyzed with high‐performance liquid chromatography (HPLC) (LC‐20A, Shimadzu Corp.) on a SIL100A column (250 × 4.6 mm, 5 μm; GL Sciences Inc.). The mobile phase was a mixture of hexane and isopropanol (99.5:0.5, v/v) at a rate of 1ml/min. *α*‐ and *γ*‐tocopherols were identified at 292 nm and 298 nm, respectively.

#### 2.5.9 Phytosterols

The content of phytosterols in oils was quantified according to Xie's method (2017) with the same modifications. Oil sample (0.2 g) (accuracy 0.0001g) and 0.5 ml of 0.5 mg/ml 5*α*‐ cholestane(internal stand) were saponified with 10 ml of 2 mol/L KOH in ethanol at 60°C for 60 min. The hexane layer was dried with anhydrous sodium sulfate and silylated using 100 *µ*l *N*, O‐Bis (trimethylsilyl) trifluoroacetamide + 1% trimethylchlorosilane (BSTFA + TMCS) at 105°C for 15 min. Then, the mixture was dissolved in 1 ml hexane for further analysis by an Agilent 6890A gas chromatography system (Agilent) equipped with a DB‐5HT column (30 m × 0.32 mm, 0.1 μm; Agilent). The flow rate of nitrogen (carrier gas) was 2.0 ml/min. The detector and injection temperature were 320°C. The oven temperature was programmed by keeping at 60°C for 1 min and increased to 310°C at a rate of 40°C/min. The temperature of 310°C was held for 10 min. The injection volume was 1 *μ*l, and the split ratio was 25:1.

### 2.6 Statistical analysis

The results were reported as mean ± standard error in triplicates. Statistical analysis was performed using a SPSS program (SPSS 20.0 for Windows, SPSS Inc.). Significances of difference between the means were determined by one‐way analysis of variance (ANOVA), using Duncan's test at a significance level of 5% (*p* < .05).

## 3 RESULTS AND DISCUSSION

### 3.1 Effect of the ratio of R134a and butane on extraction yield

**Figure 1:**
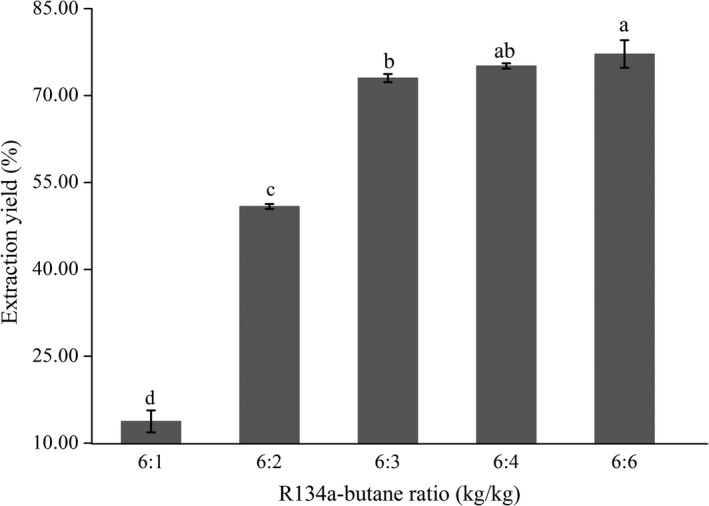
Effect of the ratio of R134a and butane on extraction yield

The impact of the ratio of R134a and butane on the extraction yield was conducted with five ratios (6:1, 6:2, 6:2, 6:4, and 6:6 (kg/kg)) and is shown in Figure 1. The results indicated that the extraction yield obviously depended on the ratio. It rose with an increase in the proportion of butane in the mixed solvent. Afterward, the yield tended to be stable when R134a and butane were in the mass ratio of 6:3 (kg/kg), that is, the ratio was 2 kg/kg. Furthermore, there were no significant differences in the yield between the ratio at 6:4 and 6:3 (kg/kg) (*p* > .05), nor between 6:4 and 6:6 (kg/kg) (*p* > .05). Therefore, the ratio at 6:3 (kg/kg) was selected as the central point of extraction ratio in the optimization experiments.

#### 3.1.1 Optimization of subcritical R134a‐n‐butane extraction conditions

Response surface methodology (RSM) is an excellent experimental design and analysis method. It is widely used in the optimization of extraction process of oil and plant functional substances (Bezerra, Santelli, Oliveira, Villar, & Escaleira, 2008). Box–Behnken experimental design is commonly used in the RSM method and suitable for the optimization experiments of three to seven factors. The relationship between independent variables (factors) and dependent variables (response values) can be approximated by multiple quadratic equations. In this study, the Box–Behnken experiment designed with three factors and three levels was adopted. A total of 17 experiments (including 12 discrete factorial points and 5 repetitive central points) were carried out. The pure error of the whole experiment was estimated by analyzing the repetitive central test points. The arrangement and results are shown in Table 2.

**Table 2:** Experimental design and results of Box–Behnken

Run	A R134a‐butane ratio (kg/kg)	B Extraction time (min)	C Extraction temperature (°C)	Extraction yield (%)
1	−1 (1)	−1 (10)	0 (35)	61.32
2	+1 (3)	−1 (10)	0 (35)	36.89
3	−1 (1)	+1 (50)	0 (35)	81.42
4	+1 (3)	+1 (50)	0 (35)	55.67
5	−1 (1)	0 (30)	−1 (25)	74.31
6	+1 (3)	0 (30)	−1 (25)	35.18
7	−1 (1)	0 (30)	+1 (45)	81.71
8	+1 (3)	0 (30)	+1 (45)	66.64
9	0 (2)	−1 (10)	−1 (25)	47.87
10	0 (2)	+1 (50)	−1 (25)	66.41
11	0 (2)	−1 (10)	+1 (45)	62.49
12	0 (2)	+1 (50)	+1 (45)	86.53
13	0 (2)	0 (30)	0 (35)	73.01
14	0 (2)	0 (30)	0 (35)	73.50
15	0 (2)	0 (30)	0 (35)	72.53
16	0 (2)	0 (30)	0 (35)	74.06
17	0 (2)	0 (30)	0 (35)	73.01

The extraction yield went from 36.89% to 86.53% depending on the extraction conditions. Further analysis by Design Expert 8.0 software found that the experimental data were fitted with the quadratic multivariate equation as following: Y (%) = 73.22–13.05A + 10.18B + 9.20C–0.33AB + 6.02AC + 1.38BC–7.88A^2^–6.52B^2^–0.88C^2^, where Y was the extraction yield; A, B, and C were R134a‐butane ratio, extraction time, and extraction temperature, respectively. Moreover, the equation illustrated the regression coefficients of the linear, quadratic, and interaction of the extraction yield. Test of significance and ANOVA for response surface quadratic model are presented in Table 3. The *F* value of the model was 388.61, and the *p* value was low 0.0001, which suggested that the model was high significant. At the same time, the *p* value of the missing item was 0.06 and not significant, indicating that the model fitted well. It was feasible to fit the relationship between the three factors and oil extraction rate by the equation. The correlation coefficients (R^2^) and Adj‐R^2^ were 0.9727 and 0.9953, respectively, which implied that the predicted values were well correlated with the actual values. The model can be used to predict the oil extraction rate of rapeseed cake. The results also showed that A, B, and C had significant effects on oil extraction yield (*p* < .01). Secondary items A^2^ and B^2^ had significant effects (*p* < .01), while C^2^ had no significant effects (*p* > .05). In addition, the absolute value corresponding to the coefficients of different factors can directly assess the influence of different factors on extraction yield (Y) (Solanki, Parikh, & Parikh, 2007). The order was R134a‐butane ratio > extraction time > extraction temperature.

**Table 3:** Test of significance and ANOVA for response surface quadratic model

Source	Sum of squares	*df*	Mean square	*F*‐value	*p*‐value
Model	3,497.50	9	388.61	377.74	<.0001
A	1,361.90	1	1,361.90	1,323.81	<.0001
B	829.47	1	829.47	806.27	<.0001
C	677.12	1	677.12	658.18	<.0001
AB	0.44	1	0.44	0.42	.5360
AC	144.72	1	144.72	140.67	<.0001
BC	7.56	1	7.56	7.55	.0301
A^2^	261.52	1	261.52	254.20	<.0001
B^2^	178.77	1	178.77	173.77	<.0001
C^2^	3.27	1	3.27	3.18	.1179
Residual	7.20	7	1.03		
Lack of fit	5.85	3	1.95	5.79	.0615
Pure error	1.35	4	0.34		
Cor total	3,504.70	16			

**Figure 2:**
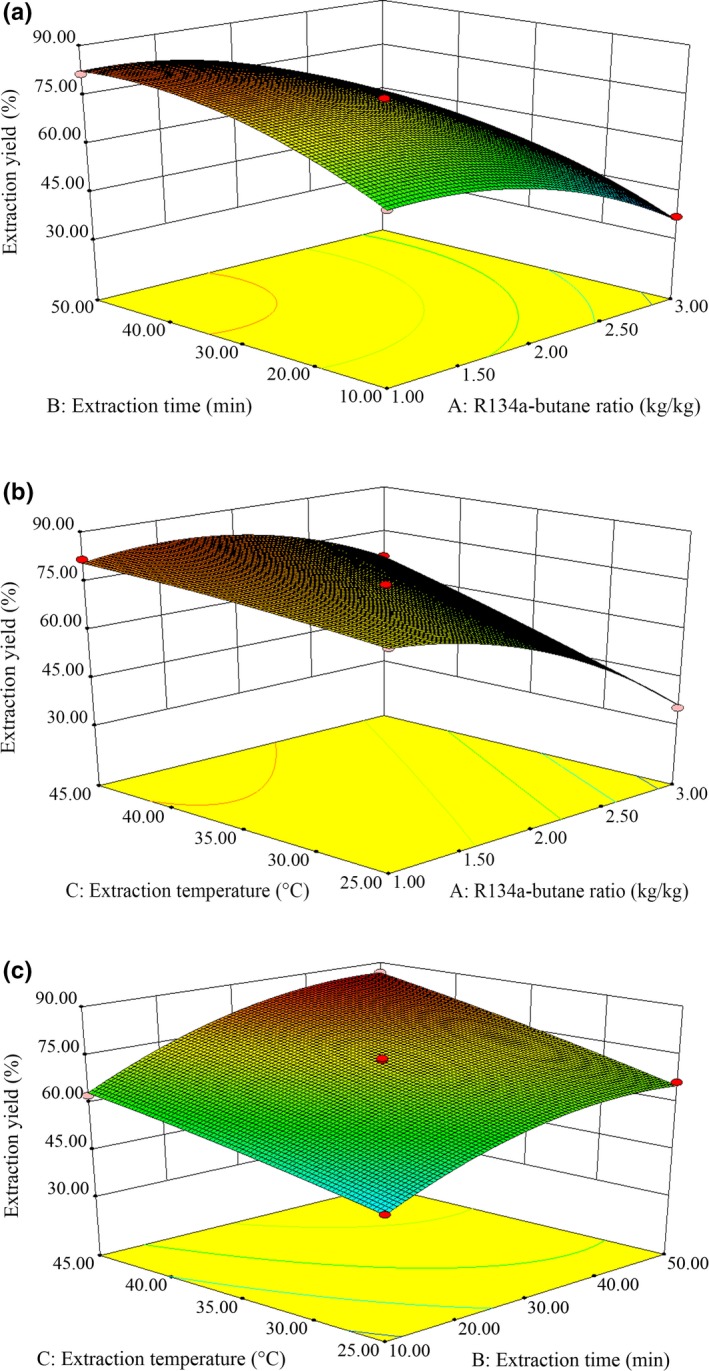
Response surface plots for the interaction of different factors. (a) R134a‐butane ratio and extraction time; (b) R134a‐butane ratio and extraction temperature; (c) extraction time and extraction temperature

The shape of contour plot can reflect the strength of interaction effect. Elliptical shape shows that two factors interact significantly, while circular shape shows the opposite (Porto, Porro, & Decorti, 2013). Contour and response surface plots are shown in Figure 2. The interaction effects between R134a‐butane ratio and extraction temperature, extraction time, and extraction temperature on oil extraction yield were significant, while that of R134a‐butane ratio and extraction time had no significant difference. The three response surfaces were convex surfaces with open‐ended downwards, which indicated that there were corresponding maximum values in the experimental range. The optimum R134a‐butane ratio, extraction time, and extraction temperature were 1.5 kg/kg, 47.99 min, and 45°C, respectively. Under the conditions, the predicted oil yield was 88.37%.

In order to verify the optimum conditions, the extraction parameters were corrected appropriately. In practice, the optimum conditions were adjusted as follows: R134a‐butane ratio was 1.5 kg/kg, the extraction time was 50 min, and the extraction temperature was 45°C. The corresponding yield was 87.76% and close to the predicted value. The corresponding yield was 87.76% and close to the predicted value. The results suggested that model was reliable and could be used to predict actual yield of rapeseed cake oil by subcritical extraction.

#### 3.1.2 Extraction yield of different methods

The extraction yield of oil from rapeseed cake obtained by supercritical CO_2_ extraction (SCO_2_E), subcritical R134a/butane extraction (SRBE), and hexane extraction (HXE) is shown in Table 4. The conventional extraction with hexane revealed the yield (74.25%) at the room pressure and 40°C for 50 min, and that of SCO_2_E was 74.18% at 28 MPa pressure and 50°C for 50 min. They were inferior to SRBE. Sun et al. (2018) also reported that the lipid extraction capacity of subcritical fluid was better than SCO_2_ and HX, and SCO_2_ and HX had no significant difference. Noteworthily, the working pressure of SCO_2_E was 28 MPa, which was much higher than that of SRBE. The severe operating conditions in this process necessitate high‐duty equipment to withstand the high pressures and the investment is expensive (Sparks, Hernandez, Zappi, Blackwell, & Fleming, 2006). Compared with SRBE, HXE has less investment in disposable fixed asset. However, its steam consumption for desolvation is 11 times higher than that of subcritical process. Therefore, subcritical extraction was more economical in terms of long‐term investment.

**Table 4:** Extraction conditions and yield of different methods

Extraction methods	Extraction pressure (MPa)	Extraction temperature (°C)	Extraction time (min)	Extraction yield (%)
SCO_2_E	28.00	50	50	74.18 ± 2.19^b^
SRBE	1.05	45	50	87.76 ± 1.09^a^
HXE	0.10	45	50	74.25 ± 1.77^b^

Note

Different letters in a column mean significant difference at the 5% level.

Abbreviations: SCO_2_E, supercritical CO_2_ extraction; SRBE, subcritical R134a/butane extraction; HXE, hexane extraction.

#### 3.1.3 Physicochemical characteristics of oils extracted by different methods

Physicochemical characteristics of rapeseed cake oils extracted by different methods are shown in Table 5. The color and viscosity of oils extracted by different methods revealed significant difference (*p* < .05). Red values of SCO_2_EO and SRBEO were 2.2 and 2.4, respectively, lower than that of nHXEO, while yellow values were higher than that of the latter, and so too of viscosity values. Acid value of nHXEO was 2.08 mg/g, which was 0.94 mg/g and 0.86 mg/g more than SCO_2_EO and SRBEO, respectively. It was due to lipase produced by endogenous or external microorganisms in rapeseed cake that has stronger hydrolytic activity in the presence of trace water. Under high‐pressure conditions of SCO_2_E and SRBE, much water was lost in rapeseed cake, and the lipase activity is inhibited. Comparatively, lipase was active in nHXE, which led to the hydrolysis of some rapeseed oil, thus increasing the acid value. The peroxide value of nHXEO was also higher, reaching 1.75 meq. O_2_/kg. SCO_2_EO and SRBEO had refractive indexes of 1.48 and 1.47, respectively, which were equal to soybean oil (1.477) and corn oil (1.473) (Nehdi et al., 2012), and higher than nHXEO. High refractive index of oil was because of high content of unsaturated fatty acid (UFA). Furthermore, saponification value of three oils had no significant difference (*p* > .05). The higher iodine value (47.82 g/100 g of oil) of SCO_2_E was by the reason of its low content of SFA.

**Table 5:** Physicochemical characteristics of oils extracted by different methods

Properties	SCO_2_EO	SRBEO	HXEO
Color			
Red	2.20 ± 0.00^c^	2.40 ± 0.00^b^	6.10 ± 0.00^a^
Yellow	32.60 ± 0.00^a^	30.50 ± 0.00^b^	18.90 ± 0.00^c^
Acid value (mg/g oil)	1.14 ± 0.01^a^	1.22 ± 0.03^b^	2.08 ± 0.12^a^
Peroxide value (meq. O_2_/kg oil)	0.89 ± 0.02^b^	0.92 ± 0.03^b^	1.75 ± 0.01^a^
Viscosity (Pa·S)	0.057 ± 0.00^b^	0.061 ± 0.00^a^	0.046 ± 0.00^c^
Refractive index	1.48 ± 0.01^a^	1.47 ± 0.00^a^	1.34 ± 0.01^b^
Saponification (mg KOH/g oil)	194.75 ± 1.78^a^	197.75 ± 3.20^a^	193.25 ± 2.49^a^
Iodine value (g/100g oil)	47.82 ± 0.11^a^	47.50 ± 0.00^b^	47.44 ± 0.00^b^

Note

Different letters in a row mean significant difference at the 5% level.

Abbreviations: SCO_2_EO, supercritical CO_2_ extraction oil; SRBEO, subcritical R134a/butane extraction oil; HXEO, hexane extraction oil.

#### 3.1.4 Fatty acid composition of oils extracted by different methods

Fatty acid (FA) composition is a major index of oil quality. The fatty acid composition of the extraction oil is shown in Table 6. In this study, eight different fatty acids were identified. The composition of fatty acids in different oils was similar, and the carbon chain of each fatty acid was 16–18. Oleic acid (C18:1) content was found to be the highest in oils (63.15%–63.84%) and it in SCO_2_EO was somewhat higher than others.

**Table 6:** Fatty acid composition of oils extracted by different methods

Fat acids (%)	SCO_2_EO	SRBEO	HXEO
Palmitic (C16:0)	4.55 ± 0.02^b^	4.50 ± 0.02^b^	4.65 ± 0.02^a^
Stearic (C18:0)	2.34 ± 0.04^b^	2.45 ± 0.00^a^	2.42 ± 0.01^a^
Oleic (C18:1)	63.84 ± 0.19^a^	63.28 ± 0.01^b^	63.15 ± 0.18^b^
Linoleic (C18:2)	19.38 ± 0.02^b^	19.22 ± 0.02^c^	19.66 ± 0.09^a^
Linolenic (C18:3)	8.00 ± 0.02^a^	7.97 ± 0.01^a^	7.81 ± 0.06^b^
Eicosanoic (C20:1)	1.34 ± 0.08^b^	1.54 ± 0.03^a^	1.43 ± 0.01^ab^
Erucic (C22:1)	0.54 ± 0.04^c^	1.05 ± 0.00^a^	0.63 ± 0.00^a^
SFA	6.89 ± 0.02^b^	6.94 ± 0.02^b^	7.07 ± 0.03^a^
MUFA^2^	65.72 ± 0.07^a^	65.87 ± 27.19^a^	65.21 ± 0.19^b^
PUFA^3^	27.39 ± 0.05^ab^	27.19 ± 0.03^b^	27.47 ± 0.15^a^
UFA^4^	93.11 ± 0.02^a^	93.06 ± 0.02^a^	92.68 ± 0.34^a^

Note

Different letters in a row mean significant difference at the 5% level.

Abbreviations: SFA, saturated fatty acids; MUFA, monounsaturated fatty acids; PUFA, polyunsaturated fatty acids; UFA, unsaturated fatty acids.

Oleic acid is an important monounsaturated fatty acid (MUFA) which exerts important effect on the prevention and treatment of cardiovascular diseases (Gu, Liu‐X, Liu‐H, Pang, & Qin, 2017). Next in much were linoleic acid (C18:2; 19.22%–19.66%) and linolenic acid (C18:3; 7.81%–8.00%), which were essential 18‐carbon unsaturated fatty acid (UFA). These fatty acids were of great significances to growth and development of human body. The results were in accordance with Jenab, Rezaei, and Emamdjomeh (2010), who also described that oleic acid was the primary fatty acid in rapeseed oil (over 60% of total fatty acids), followed by linoleic acid and linolenic acid at approximate percentages (17.2% and 8.0%, respectively).

Statistical analysis showed that it had significant differences in fatty acid composition (*p* < .05) but no significant difference in the total content of UFA (*p* > .05) among the three extraction oils. Content of UFA was dominated in all samples (92.68%–93.11%), particularly similar to Pederssetti et al. (2011). The MUFA content was high than 65%. Except for oleic acid, which accounted for ~62%, eicosanoic acid (C20:1) and erucic acid (C22:1) presented lower than 1.6%. The polyunsaturated fatty acid (PUFA) was mainly including linoleic acid and linolenic acid, between 27.19% and 27.47%. Saturated fatty acid (SFA) such as palmitic (C16:0) and stearic acid (C18:0) contributed 6.89%–7.07%, and it was comparable to that of Cvjetko et al. (2012) (7.06%–7.07%). Overall, fatty acids were extracted nonselectively by different extraction methods and no isomerization or oxidation of fatty acids occurred under the extraction conditions.

#### 3.1.5 Phospholipids and Phospholipid compositions of oils extracted by different methods

The presence of phosphatides (PLs) is undesirable because they are harmful to color, flavor, foaming of the edible oil, which is not conducive to the consumption and sale. In addition, phospholipids often exist together with metal ions by chelation, which can act as catalysts for lipid oxidation, leading to lipid oxidation and rancidity (Goh, Khor, & Gee, 1982).

The results in Table 7 showed that there were significant differences in the PLs and PL composition content of the oil extracted by the three processes. PLs in SCO_2_EO were lower than the detection limit. The result concurred with Wan, Zhang, Huang, and Guo (2018), who reported extremely low phospholipids were detected in canola oil extracted by supercritical carbon dioxide. It was ascribed to CO_2_ is a nonpolar molecule and only suitable for extracting nonpolar or weak polar substances (such as phospholipid) (Zaidul, Norulaini, & Omar, 2006). The content of PLs in HXEO was the highest, reaching 25.78 mg/g, about 8.56 times of SRBEO. Xu et al. (2016) also described PLs were more easily extracted in hexane than in subcritical and supercritical fluid. In terms of PL compositions, only phosphatidylcholine (PC) and phosphatidylinositol (PI) existed in SRBEO and HXEO. The content of PI in oils was 1.22–9.45 mg/g, accounting for 59.25%–73.05% in PLs, whereas PC presented a relatively lower percentage (26.95%–40.75%). Moreover, with the increase in PL content, the proportion of them increased. These results indicated that the extracted oil by subcritical R134a/butane had lower phosphatides and phosphatide compositions.

**Table 7:** PLs and PL compositions of oils extracted by different methods

Extraction methods	PLs (mg/g)	PC (mg/g)	PE (mg/g)	PI (mg/g)
SCO_2_EO	ND	ND	ND	ND
SRBEO	3.01 ± 0.14^b^	0.45 ± 0.18^b^	ND	1.22 ± 0.16^b^
HXEO	25.78 ± 0.31^a^	6.50 ± 0.16^a^	ND	9.45 ± 0.27^a^

Note

Different letters in a column mean significant difference at the 5% level.

Abbreviations: PLs, phospholipids; PC, phosphatidylcholine; PE, phosphatidylethanolamine; PI, phosphatidylinositol; ND, not detected.

#### 3.1.6 Minor components of oils extracted by different methods

2,6‐dimethoxy‐4‐vinylphenol (vinylsyringol), otherwise known as canolol, is one of the main phenols in rapeseed. It can be formed by decarboxylation of the sinapic acid and accounts for 70%–85% of the total free phenolic acid (Koski. Pekkarinen, Hopia, Kristiina, & Marina, 2003; Wakamatsu et al., 2005). Canolol has been proved to reveal good antioxidative and antimutagenic properties in body (Kuwahara et al., 2004). During rapeseed pressed, only a small proportion of phenol is transferred to the crude oil, and most of them remain in the cake. Therefore, rapeseed cake is a great resource of canolol. Canolol has certain polarity and is easy to combine with proteins or polysaccharides to form stable compounds by hydrogen bonds and hydrophobic bonds. Different extraction solvents would directly affect the break of chemical bond and the release of canolol.

The results of canolol content in oil extracted by three solvents with different polarities are obtained in Table 8. The detailed data of SCO_2_EO, SRBEO, and HXEO were 95.82, 118.51, and 131.85 mg/100g, obviously higher than Siger, Michalak, and Rudzińska (2016), who produced oil using hexane from rapeseed expeller cake, but in accordance with Wan et al. (2018), who demonstrated that canolol of oil extracted by SCO_2_ was lower than others. The effect of different solvents on the PL content performed a similar trend to that on canolol. Wan (2017) calculated the polarity of R134a and butane was 0.7 and 0, respectively, while that of canolol, PC, and PI was 0.5, 0.2, and 0.4, respectively. Hence, the binary mixture of R134a and butane was beneficial to the extraction of canolol and PLs.

**Table 8:** Minor components of oils extracted by different methods [Correction added on 4 October 2019, after first online publication: The Table 8 has been updated.]

Minor components	SCO_2_EO	SRBEO	HXEO
Canolol (mg/100g)	95.82 ± 1.14^c^	118.51 ± 2.85^b^	131.85 ± 0.83^a^
*β*‐Carotene (ug/100g)	221.65 ± 12.57^b^	357.21 ± 16.62^a^	328.14 ± 19.59^a^
Tocopherols (mg/100g)			
*α*‐Tocopherol	40.36 ± 1.38^a^	42.10 ± 0.97^a^	35.88 ± 1.33^b^
*γ*‐Tocopherol	54.68 ± 0.37^a^	49.76 ± 1.66^ab^	46.84 ± 2.35^b^
Total	95.04 ± 1.75^a^	91.86 ± 2.63^a^	82.72 ± 3.68^b^
Phytosterols (mg/100g)			
Brassicasterol	173.69 ± 3.54^a^	150.21 ± 1.84^b^	148.94 ± 1.93^b^
Campesterol	414.38 ± 7.04^a^	386.59 ± 4.64^b^	367.48 ± 5.3^c^
Sitosterol	591.40 ± 11.39^a^	560.19 ± 5.92^b^	531.38 ± 5.22^c^
Total	1,179.46 ± 21.97^a^	1,096.99 ± 12.40^b^	1,047.80 ± 12.5^b^

Note

Different letters in a row mean significant difference at the 5% level.

#### 3.1.7 *β*‐Carotene, tocopherols, and phytosterols of oil extracted by different methods

*β*‐Carotene protected lipids from free radical oxidation by reacting with peroxide free radicals, thus inhibiting the proliferation of lipid and promoting the termination of oxidation chain reaction (Sun, Pang, Ye, Lü, & Li, 2009). *β*‐Carotene is also an effective quenching agent while inhibiting singlet oxygen (Yang et al., 2013). As shown in Table 8, the amount of *β*‐carotene in SRBEO and HXEO was at an equal level (*p* > .05) and was significantly higher than that in SCO_2_EO (221.65 μg/100 g). This owed to the lower solubility and/or mass transfer rate of *β*‐carotene in SCO_2._ These results indicated that SRBEO had better quality than SCO_2_EO and HXEO due to a higher amount of *β*‐carotene.

Tocopherols, also known as vitamin E, are powerful natural antioxidants that could effectively inhibit lipid oxidation. The tocopherols of the extraction oils by different methods are presented in Table 8. The tocopherols in rapeseed oil were mainly comprised of *α*‐tocopherol (35.88–42.10 mg/100 g) and *γ*‐tocopherol (46.84–54.68 mg/100 g), and the content of latter is comparatively higher. The total tocopherols in SRBEO and SCO_2_EO had no significant difference (*p* > .05), which reached the maximum (>90 mg/100 g). While the *α*‐tocopherol of SRBEO was slightly higher than that of SCO_2_EO, the *γ*‐tocopherol showed lower than SCO_2_EO. Therefore, we inferred that besides solvent polarity, other factors can also affect the extraction of tocopherols.

Phytosterol is a class of chemical compounds with cyclopentane and phenanthrene as its skeleton, which has physiological functions such as reducing the incidence of heart disease, anticancer, and immune regulation (Yang et al., 2013). As the results of phytosterols in oils are demonstrated in Table 8, it could be clearly known that the contents of total phytosterols varied from 1,047.80 mg/100 g to 1,179.46 mg/100 g, and the order was SCO_2_EO > SRBEO>HXEO. Three phytosterols were detected in this study, and sitosterol was the dominant phytosterols (50.14% ‐ 51.07% of total phytosterols), followed by campesterol (35.07% ‐ 35.36%) and brassicasterol (13.69%–14.73%). These results were consistent with the results reported by Hamama, Bhardwaj, and Starner (2003), where the percent of them were 47.2%–50.6%, 29.8%–34.6%, and 10.1%–17.2%, respectively. Moreover, the contents of brassicasterol, campesterol, and sitosterol in extraction oils were in the same order as those of total phytosterols. The extraction ability of subcritical R134a/butane was weaker than that of SCO_2_, but stronger than that of hexane.

## 4 CONCLUSIONS

Results of response surface methodology indicated that the peak performance of subcritical R134a/butane extraction for rapeseed cake oil was carried out with the parameters as follows: R134a‐butane ratio of 1.5 kg/kg, extraction temperature of 45°C, and extraction time of 50 min. The extraction yield was significantly higher than SCO_2_E and HXE. Evaluating the quality of extraction oils found that the fatty acid composition in all oils was almost the same, but the contents of other minor components were significantly different. Subcritical R134a/butane extraction oil owned the highest tocopherols and *β*‐carotene, higher canolol and phytosterols but less phospholipids. Overall, subcritical R134a/butane extraction was an efficient extraction method for rapeseed cake, and it is potential to be an alternative to supercritical CO_2_ extraction and traditional hexane extraction.

## CONFLICT OF INTEREST

The authors declared that we had no conflict of interest.

## ETHICAL APPROVAL

The study did not include any animal or human tests.
